# Viral-mediated transduction of auditory neurons with opsins for optical and hybrid activation

**DOI:** 10.1038/s41598-021-90764-9

**Published:** 2021-05-27

**Authors:** Rachael T. Richardson, Alex C. Thompson, Andrew K. Wise, Elise A. Ajay, Niliksha Gunewardene, Stephen J. O’Leary, Paul R. Stoddart, James B. Fallon

**Affiliations:** 1 The Bionics Institute, East Melbourne, VIC 3002 Australia; 2 Department of Surgery (Otolaryngology), University of Melbourne, The Royal Victorian Eye and Ear Hospital, East Melbourne, VIC 3002 Australia; 3 Medical Bionics Department, University of Melbourne, Parkville, VIC Australia; 4 Faculty of Science, Engineering and Technology, Swinburne University of Technology, Hawthorn, VIC 3122 Australia; 5 Department of Biomedical Engineering, University of Melbourne, Parkville, VIC Australia

**Keywords:** Gene therapy, Cochlea, Biomedical engineering

## Abstract

Optical stimulation is a paradigm-shifting approach to modulating neural activity that has the potential to overcome the issue of current spread that occurs with electrical stimulation by providing focused stimuli. But optical stimulation either requires high power infrared light or genetic modification of neurons to make them responsive to lower power visible light. This work examines optical activation of auditory neurons following optogenetic modification via AAV injection in two species (mouse and guinea pig). An Anc80 viral vector was used to express the channelrhodopsin variant ChR2-H134R fused to a fluorescent reporter gene under the control of the human synapsin-1 promoter. The AAV was administered directly to the cochlea (n = 33) or posterior semi-circular canal of C57BL/6 mice (n = 4) or to guinea pig cochleae (n = 6). Light (488 nm), electrical stimuli or the combination of these (hybrid stimulation) was delivered to the cochlea via a laser-coupled optical fibre and co-located platinum wire. Activation thresholds, spread of activation and stimulus interactions were obtained from multi-unit recordings from the central nucleus of the inferior colliculus of injected mice, as well as ChR2-H134R transgenic mice (n = 4). Expression of ChR2-H134R was examined by histology. In the mouse, transduction of auditory neurons by the Anc80 viral vector was most successful when injected at a neonatal age with up to 89% of neurons transduced. Auditory neuron transductions were not successful in guinea pigs. Inferior colliculus responses to optical stimuli were detected in a cochleotopic manner in all mice with ChR2-H134R expression. There was a significant correlation between lower activation thresholds in mice and higher proportions of transduced neurons. There was no difference in spread of activation between optical stimulation and electrical stimulation provided by the light/electrical delivery system used here (optical fibre with bonded 25 µm platinum/iridium wire). Hybrid stimulation, comprised of sub-threshold optical stimulation to ‘prime’ or raise the excitability of the neurons, lowered the threshold for electrical activation in most cases, but the impact on excitation width was more variable compared to transgenic mice. This study demonstrates the impact of opsin expression levels and expression pattern on optical and hybrid stimulation when considering optical or hybrid stimulation techniques for neuromodulation.

## Introduction

Neuromodulation by electrical stimulation is the current standard for managing conditions such as hearing loss and movement disorders. Approximately 700,000 people worldwide are surgically fitted with a cochlear implant which provides electrical stimulation to auditory neurons (spiral ganglion neurons; SGNs) for hearing rehabilitation. While the cochlear implant enables recipients to understand speech in many cases, outcomes are varied and speech comprehension with even moderate competing background noise is poor, as is appreciation of music and understanding of tonal languages^1–4^. One of the reasons for poor outcomes is the inherent spread of electrical current which radiates out from the stimulating electrodes. As a result, a broad population of SGNs can be activated by a single electrode which reduces the number of perceptually independent channels^5–10^ and prohibits the relay of precise sub-millisecond timing information (temporal fine structure) to specific positions in the cochlea. This is in contrast to the normally sharp tuning provided by the cochlear sensory hair cells to specific frequencies that map tonotopically along the cochlear axis. Improved spectral precision of neural activation in the cochlea has been demonstrated using current focussing techniques^11–14^, but the clinical benefit has been limited as they cannot deliver fine temporal structure for simultaneous multi-channel stimulation^11,15^.

Optical stimulation is a paradigm-shifting approach to cochlear neural modulation and can be applied using near infrared light^16–20^ or optogenetic techniques^21–27^. For infrared neural stimulation, the requirement for high energy pulses and the risk of thermal damage have impeded the clinical development of this technique^28^. Furthermore, we and others could not detect neural responses in animals with profound loss of hair cells^29–32^. With optogenetic techniques, the neuron is genetically modified with a light-gated ion channel (opsin) to make it responsive to light in the visible spectrum^27^. Optogenetic techniques use up to 75 times less power to activate neurons compared to near infrared light^21,24,33^ which would significantly reduce unwanted tissue heating that may occur at high stimulation rates^28^. The spread of cochlear excitation achieved with optical stimulation of opsin-modified SGNs can be significantly reduced when compared to electrical stimulation, nearing that achieved with acoustic stimulation in animals with normal hearing^26^. Furthermore, the requirement for high temporal fidelity of the auditory system can also be met with the use of ultrafast opsin variants such as Chronos or f-Chrimson, thus allowing neurons to follow stimulation at rates in the order of 200 Hz^22,25^, but still with a high power budget. Alternatively, hybrid stimulation may be used, in which a subthreshold electrical stimulus is applied together with the sub- or supra-threshold optical stimulus^34,35^, thus exploiting the high rate properties of electrical stimulation while using more energy efficient slower opsins to raise the excitability of neurons and reduce the spread of activation.

One of the key prerequisites for application of optogenetic techniques in auditory activation is a method for genetic modification of SGNs that is reliable, consistent, clinically relevant and safe. Proof of concept studies have benefitted from transgenic mice for specific, consistent and ubiquitous expression of opsins in SGNs^24,34,35^ but there is a need to validate these results using clinically translatable transduction strategies such as viral-based gene transfer for any future clinical application of this technology. The validity of optogenetic activation of virally transduced SGNs has been tested using embryonic and neonatal AAV injection techniques in mice as transduction rates are typically higher in juvenile mice compared to adult mice, with expression in up to 80% of SGNs reported^22–25^. However, at this age the auditory system is still developing and transduction efficiency in adult mice drops off dramatically^36^. In the adult gerbil, intrascalar injection of a viral vector resulted in virtually no transduction^21^. A pressure injection directly into the modiolus (the central axis of the cochlea containing the SGNs) of adult gerbils improved transduction, with an average 30% transduction of SGNs in all turns of the spiral-shaped cochlea in gerbils that had a functional response to optical stimulation (approximately half of those in the study)^21,26^. However, this injection technique is more traumatic than injection into the cochlear fluid-filled cavities and there was a loss of a quarter of the SGN population^21^.

Using a transgenic mouse model, it was shown that SGNs with twofold greater expression of opsins had increased photosensitivity at the cellular level and for optical evoked auditory brainstem responses (oABRs)^37^. For animals transduced by AAV injection, oABRs could be recorded from animals that had as little as 10% of the SGN population transduced and the oABR amplitude correlated positively with the percentage of transduced SGNs^21^. Thus, transduction efficiency is a key factor for optimum performance of optical stimulation.

In this study, we compare AAV injection approaches for transducing SGNs with a channelrhodopsin variant ChR2-H134R fused to a reporter gene. The viral vector was injected directly into the cochlea of mice and guinea pigs through the round window (a natural membrane-covered opening) with or without fenestration of the semi-circular canal to allow fluid flow through the cochlea^38^. Indirect injection via the posterior semi-circular canal was also used. After at least 20 days post-injection, optical and electrical stimuli were applied individually or as hybrid stimuli to the cochlea of the mice while recording multiunit activity from the inferior colliculus of the auditory midbrain. Opsin expression was then examined histologically to examine the influence of opsin expression levels on optical and/or electrical activation in the cochlea. A key finding of the study was a significant influence of the density and pattern of opsin expression in the SGN population on both optical-only and hybrid activation. Reproducible, efficient and effective techniques for transducing SGNs with opsins will be essential for clinical application of optogenetic neural modulation.

## Methods

### Animals

C57BL/6 mice were purchased from the Walter and Eliza Hall Institute (Melbourne, Australia). ChR2-H134R transgenic mice were derived by breeding COP4*H134R/EYFP mice (Jax strain 012569: B6;129S-Gt(ROSA)26Sor^tm32(CAG-COP4*H134R/EYFP)Hze/J^, backcrossed onto a C57BL/6 background) with Cre-parvalbumin mice (Jax strain 008069: B6;129P2-Pvalb^tm1(cre)Arbr^) and thus carry one allele for ChR2-H134R-EYFP. Dunkin Hartley guinea pigs were purchased from St Vincent’s Hospital Biological Resource Centre (Melbourne, Australia). The use and care of the experimental animals in this study were approved by St Vincent’s Hospital Animal Ethics Committee (#14-028, #16-007, #18-003, #20-004, #18-396) following the Guidelines to Promote the Wellbeing of Animals used for Scientific Purposes (2013), the NHMRC Code for Care and Use of Animals for Scientific Purposes (8th edition, 2013) and the Prevention of Cruelty to Animals Amendment Act (2015). A total of 37 injected mice (11 neonatal, 23 adult, 3 juvenile), 4 transgenic mice and 6 guinea pigs were used in this study.

### AAV injection

The AAV vector AAVAnc80L65-hSyn-ChR2(H134R)-EYFP was purchased from the Vector and Genome Engineering Facility, Children’s Medical Research Institute, NSW, Australia (Addgene plasmid #26973). The humanized ChR2 with H134R mutation is fused to EYFP and driven by human Synapsin I promoter for optogenetic activation. The vector was provided in 10 mM phosphate, 50 mM NaCl, 0.001% Pluronic F-68, pH 7.4 (9.23e11 vg/mL). Fast green dye (final concentration 0.25% (w/v); Sigma-Aldrich, MO, USA, Cat# F7252) was added to the viral suspension to aid the visualisation of the injection. Age range at the time of injection was 2–139 days.

All animals were injected unilaterally. All procedures were performed under general anaesthesia: Gaseous anaesthesia (1–2% isofluorane mixed with oxygen delivered at a flow rate of 1.5 L/min) for juvenile and adult mice, injectable anaesthesia (60 mg/kg ketamine, 4 mg/kg xylazine) for adult guinea pigs or hypothermia for neonatal mice followed by recovery on a warm heat pad. With the exception of neonates, local anaesthesia (1–2% lignocaine hydrochloride) was applied at each surgical site, respiration rate was monitored during anaesthesia and a heating pad was used to maintain core body temperature.

***Neonatal mouse*** (2–5 days old; n = *11; 4 males, 4 females, 3 undetermined):* Following a post-auricular incision, the adipose and muscle layers were dissected to reveal the bulla. The bulla was pierced with a 25G needle. The bulla opening was expanded with angled forceps. The round window membrane (RWM) was identified and pierced with a pulled borosilicate pipette and the perilymph was wicked away until there was minimal efflux. A bevelled pulled borosilicate pipette containing 1 µL of the virus suspension was inserted. The virus was injected slowly by hand via a Hamilton syringe. The pipette was withdrawn and the RWM was quickly sealed with fascia or adipose tissue. The tissue layers were returned to their original position and the skin layers were superglued or sutured. The pup was warmed, rubbed with nesting material and returned to the mother.

***Juvenile*** (13–15 days old; n = *3; undetermined sex) and adult mouse (24–101 days old; n* = *13; 12 males, 1 female) via RWM: *A similar surgical approach was used as per neonatal mice with the following changes: A #11 blade was used to hand-drill through the bulla. The bevelled borosilicate pulled pipette containing the virus suspension was inserted through the RWM via a micromanipulator. Following injection, the hole in the bulla was sealed with muscle tissue and the wound was closed with sutures.

***Adult mouse injection via RWM with semi-circular canal fenestration*** (25–45 days old; n = *6; 2 males, 4 females):* The surgical approach was identical to the juvenile/adult RWM approach with the following additional steps: the muscle overlying the posterior semi-circular canal was carefully dissected from the bone and the posterior semi-circular canal was opened with a slow speed 0.5 mm diameter diamond burr until perilymph efflux was observed. During injection through the pierced RWM, perilymph was periodically wicked from the semi-circular canal until efflux of the green dye of the viral suspension was visualised at the semi-circular canal fenestration site. Muscle tissue was placed on the canalostomy site as well as the RWM prior to withdrawal of the injecting pipette.

***Adult mouse injection via semi-circular canal*** (76–139 days old; n = *4; 3 female, 1 male):* The posterior semi-circular canal was accessed and drilled as described above, without accessing the cochlea. A bevelled pulled borosilicate glass pipette was positioned in the semi-circular canal and sealed in place with a quick-drying silicone sealant. Four microliters of the viral suspension was injected via an injection controller over 3 min. Muscle tissue was placed on the canalostomy site after withdrawal of the injecting pipette.

***Adult guinea pig injection with semi-circular canal fenestration*** (57–64 days old; n = *6; 3 male, 3 female):* A post-auricular incision was made and the underlying muscles were gently separated to expose the tympanic bulla. The bulla was opened with a #14 diamond burr allowing an oblique view of the RWM. The ventral semi-circular canal was opened with a 0.5 mm diamond burr until perilymph efflux was visible. The RWM was pierced with a bent 25G needle. In two animals, the modiolus was also drilled with the diamond burr for access to the SGNs. A pulled flexible polypropylene cannula connected to a Hamilton syringe was inserted into the RWM (against the additional modiolus drill site where applicable) to inject 3 µL of prepared viral suspension. The virus was injected slowly by hand. After injection the RWM and semi-circular canal sites were sealed with Quickseal glue. The tissue layers were returned to their original position and closed with 3–0 vicryl sutures.

### Response data acquisition and analysis

At a minimum of 20 days post-injection, a subset of AAV-injected C57BL/6 mice, ChR2-H134R-EYFP transgenic mice or AAV-injected guinea pigs were placed under gaseous anaesthesia to record multiunit responses from the inferior colliculus in response to electrical, optical or hybrid stimuli. Body temperature was maintained at 37 °C with a heat pad, respiration was monitored and 1–2% lignocaine hydrochloride local anaesthesia was applied to each surgical site. The injected cochlea was exposed as described for AAV injection, removing any scar tissue from the previous surgery to reveal the entire cochlea. In some cases the mouse stapedial artery was cauterised with a bipolar electrocautery. A cochleostomy was made in the mid-apical region of the cochlea and the RWM was perforated with a metal probe. Animals were acutely deafened by applying 8–10 drops of a 10% (w/v) neomycin solution in PBS to the opened RWM while aspirating from the apical cochleostomy site (mice) or oval window (guinea pigs). This technique has been previously shown to result in near wipe-out of inner hair cells, at least in the basal turn of the cochlea^35^. The surgical site was then temporarily plugged with a saline-soaked cotton ball while the inferior colliculus was exposed. Animals were placed in a stereotaxic frame (David Kopf Instruments). A craniotomy was performed in the region of the intersection of the parietal and interparietal bones contralateral to the injected cochlea. The dura mater was gently removed to reveal the dorsal surface of the inferior colliculus for mice. Exposure of the inferior colliculus in guinea pigs required removal of brain tissue via gentle suction.

The stereotaxic frame was rotated to allow visualisation of the RWM and insertion of a 105 µm core optical fibre (125 μm silica cladding, 3 mm PVC jacket and numerical aperture of 0.22; Thorlabs, NJ, USA) bonded with cyanoacrylate to a 25 µm diameter 90/10 Platinum/Iridium wire insulated up to 0.1 mm of the tip. The distal end of the optical fibre was bare fibre. The optical fibre/platinum/iridium wire combination was inserted up to 1–2 mm into the basal turn of the cochlea via the round window (approximately following the path of the turn) or 0.5–1 mm into the apical turn of the cochlea via a cochleostomy (pointing towards the modiolus).

A single shank silicon substrate multi-channel recording array (NeuroNexus Technologies, MI, USA) with 50 µm electrode spacing was mounted in a Microdrive positioner (David Kopf Instruments, USA), positioned at the surface of the inferior colliculus and slowly advanced along the dorsal–ventral axis of the inferior colliculus while monitoring responses at the tip electrode to optical or electrical stimulation. When the recording array was within the central nucleus of the inferior colliculus, a 1% (w/v) agar solution was applied to the surface of the inferior colliculus and around the recording array to provide stability for neural recordings. The recording array was referenced via an internal reference that was kept in electrical contact with the brain via conductive agar and with grounding through a needle in the right axillary.

Stimulus waveforms were generated by an in-house purpose-built multichannel stimulator controlled by custom software implemented in Igor Pro (Wavemetrics, Portland, OR). Light was delivered to the cochlea via a custom-built solid state 488 nm laser (OptoTech, Melbourne, Australia) coupled to the optical fibre via an FC connector. Light was presented as 1, 5 or 10 ms single pulses at 0–50 mW light intensities. Irradiance was calibrated at a distance of 5 mm from the fibre tip with a Fieldmaster power meter and LM10 power meter head (Coherent, Santa Clara, CA). Laser power is presented logarithmically in decibels relative to 1 µW which is given by ${P}_{dB}=10 \times {\mathrm{log}}_{10}(\frac{P}{{10}^{-6}})$. Electrical stimulation was also provided to the cochlea via the platinum/iridium wire. A stainless steel needle placed subcutaneously in the left side axillary region of the mouse was used as the remote return electrode. Electrical current was delivered in Current Levels ‘CL’, where current in µA is given by: $I=17.5\times {100}^\frac{CL}{255}$. Biphasic electrical pulses (100 µs/phase with 50 µs interphase gap) were presented at sub- and suprathreshold levels (20–120% of threshold) with the start of each pulse delayed by up to 1.75 ms relative to the start of the optical pulse.

Multi-unit activity was processed using customised spike detection scripts in Igor Pro (Wavemetrics, Portland, OR) as per^35^. A level of four times the root mean square for each recording channel was used for threshold crossings to detect spikes. Spike counts were generated from a 5–40 ms window post stimulus and converted to neural response strength by normalising between spontaneous activity and maximum driven spike rate. Neural response strength across the array was colour-coded to display response images, with electrode number of the recording array on the x-axis and the stimulus intensity on the y-axis. A spatial tuning curve (STC) was generated for specific stimulation sites. Threshold was determined for each condition, defined as the lowest stimulus intensity to elicit a normalised spike rate of 0.3^39^. The recording site with the lowest threshold was defined as the best recording site. For hybrid stimulation, the electrical stimuli were presented at levels above and below threshold while optical power was held at various levels below optical threshold. For analysis of hybrid data, the highest subthreshold optical power was used.

The discrimination index (*d’*) was used to quantify the growth in neural response with increasing stimulus intensity at each recording site. At the best recording site, the value of d’ was cumulated across increasing stimulus levels above threshold. The widths of the STCs were measured at cumulative d’ = 1 and d’ = 2 above threshold for comparison between modalities. Difference plots were generated by obtaining the electrical data from an electrical only stimulation trial and optical data from a hybrid trial at 0 current level (i.e. virtually zero charge). These were subtracted from the hybrid run to show where responses were greater than the sum of the two.

### Histology and immunohistochemistry

Mice were terminated after an average of 32 ± 2 days (S.E.M.) after cochlear injection (range 20–82 days). Cochleae and semi-circular canals were extracted and fixed in 10% neutral buffered formalin (NBF) for 2 h. Guinea pigs were given a lethal dose of pentobarbitone at 20–42 days after injection and intracardially perfused with 0.9% (w/v) saline containing 0.1% (w/v) heparin sodium and 0.025% (w/v) sodium nitrite followed by NBF. Dissected cochleae and semi-circular canals were post-fixed in NBF for a further 2 h. Following fixation, cochleae were rinsed in PBS and decalcified in 5% (w/v) ethylenediaminetetraacetic acid (EDTA) over 2–3 days for mouse cochleae or 10% EDTA (w/v) 1–2 weeks for guinea pig cochleae. The cochleae were frozen in optimal cutting temperature compound and sectioned through the mid-modiolar plane at 12 µm and placed onto Superfrost slides. Slides were stored at -20 °C until use.

Frozen sections were thawed at room temperature, incubated in PBS for 10 min, permeabilised in PBS containing 0.2% (V/V) Triton X-100 (Sigma-Aldrich, St Louis, MO) and blocked in PBS containing 10% goat serum (Sigma-Aldrich, St Louis, MO) and 0.2% (V/V) Triton X-100 and for 1–2 h. A mouse monoclonal antibody to tubulin beta 3 (Tuj1; BioLegend, 1:500) or the C-terminus of ChR2 (#651180, Progen, Heidelberg, Germany, 1:200) was applied to the sections for 2 h, diluted in blocking buffer. After washing the sections in PBS over 30 min, a secondary antibody was applied to the sections for 2 h (AlexaFluor goat anti-mouse 594 diluted 1:400 in blocking buffer; Life Technologies, Carlsbad, CA). Sections were again washed in PBS over 30 min prior to the application of mounting media and coverslips.

Average data from at least two mid-modiolar sections per cochlea were used to assess ChR2-H134R-EYFP expression in SGNs. Images were captured using a Zeiss Axioplan fluorescence microscope (Carl Zeiss, Jena, Germany) using identical lighting conditions for each image. ImageJ software (https://imagej.nih.gov/ij/, 1997–2018) was used to determine the proportion of EYFP-positive SGNs by counting the number of EYFP-positive cells within Rosenthal’s canal and expressing this as a percentage of the total number of Tuj1-positive SGN cell bodies counted within the same area. This was performed in the hook-basal turn region (termed basal turn), middle turn and apical turn by a researcher who was blinded to the treatment group. Injected and contralateral cochleae were analysed for each animal. Thresholding was used to assist with YFP-positive cell identification using ImageJ default settings and manually adjusting the threshold until the bony modiolus was not detected.

### Statistics

Statistical analysis of ChR2-H124-EYFP expression was done by initially pooling data across cochlea regions and assessing the effectives of different routes of administration and age via a Kruskal–Wallis One way Analysis of Variance on Ranks test with Dunn’s method for post-hoc analysis, due to the non-normal distribution of the expression data (driven by the zero expression levels in some groups). Regional expression was assessed via two-Way RM ANOVA (Region, Side) in the Neonate RWM group with Holm-Sidak method for pos-hoc analysis and one-Way RM ANOVA in the injected cochlea of the Adult RWM-F group. Statistical analysis of spatial tuning curve widths was done via two-Way RM ANOVA (Mode, Region). For all ANOVAs normality was assessed via Shapiro–Wilk testing and equal variance via Brown-Forsythe.

### Ethical approval

The study was carried out in accordance with the ARRIVE guidelines.

## Results

### Viral-based ChR2-H134R gene delivery to SGNs

**Figure 1:**
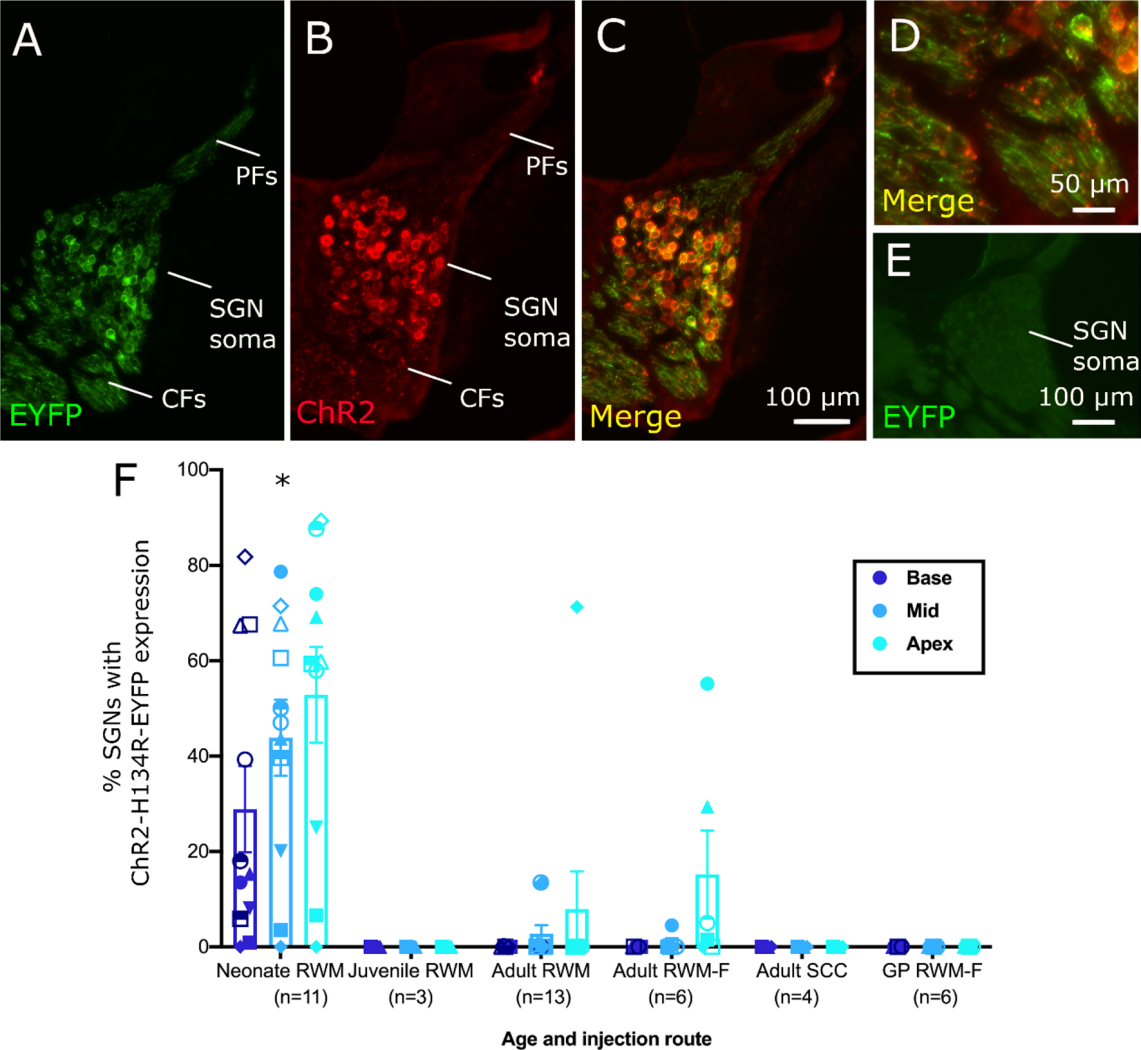
Viral-mediated transduction of SGNs with opsins. (**A**) Expression of EYFP reporter gene in the middle turn of a cochlea from a C57BL/6 mouse injected at a neonatal age. EYFP expression was detected in SGN central fibres (CFs), soma and peripheral fibres (PFs) as far as the synapse with the inner hair cells. (**B**) The same cochlear section labelled with a ChR2 antibody with notably lower expression in PFs and CFs compared to the SGNs (**C**) Merged image of EYFP expression (green) and ChR2 antibody labelling (red). (**D**) Magnified area from central nerve showing punctate ChR2 antibody labelling. (**E**) Cochlear section from an Anc80-injected mouse that had no detectable EYFP expression. (**F**) The overall percentage of ChR2-H134R-EYFP transduced SGNs in C57BL/6 mice or guinea pigs (final category) injected via different routes and at different ages. The neonatalC57BL/6 mouse injection group had the highest percentage of transduced SGNs (*p < 0.05 compared to all other groups, Kruskal–Wallis) and the highest proportion of successful transductions overall (Error bars show the standard error of the mean). Scale bar in C applies to images A-C. (SGN = spiral ganglion neuron, EYFP = yellow fluorescent protein; ChR2 = channelrhodopsin-2; RWM = round window membrane; F = fenestration of semi-circular canals; SCC = semi-circular canals; GP = guinea pig). Minor image processing (brightness) was applied equally to the EYFP channel across images for representation purposes.

Following localised Anc80 virus injection into the cochlea of C57BL/6 mice or guinea pigs, the EYFP reporter gene was used to assess the expression of ChR2-H134R-EYFP in cochlear tissue. EYFP was detected in SGNs (Fig. 1A) and, for adult mouse injections, also in inner hair cells (not shown). The EYFP reporter was confirmed to co-localise with ChR2-H134R using an antibody to ChR2 (Fig. 1B), although the antibody distribution was stronger and more uniform in the SGN cell body in contrast to the peripheral and central fibres where the binding of the antibody was more punctate compared to EYFP expression (Fig. 1B-D). As the ChR2-H134R-EYFP is a fused protein, the disparity in EYFP signal and the ChR2 antibody signal may be the result of access of the ChR2 antibody to the heavily myelinated neurons. A cochlear section from an injected mouse that was negative for EYFP expression is shown in Fig. 1E. There was no apparent influence of opsin expression on overall SGN survival with a paired t-test returning no significant difference between SGN density on the injected side (3224 ± 176 SGNs/mm^2^) and the non-injected side (2743 ± 320 SGNs/mm^2^) in the basal turn (p = 0.43, n = 18) and similar results in the middle (p = 0.52) and apical turns (p = 0.53).

Three surgical approaches to intracochlear Anc80 injection were tested: RWM injection (‘RWM’), RWM injection with fenestration of the posterior semi-circular canal (‘RWM-F’) and injection into the posterior semi-circular canal (‘SCC’). We also tested different age sub-groups and different species within some of these methods. A successfully transduced cochlea was defined as displaying any EYFP-positive SGN in any turn. There was large variability in ChR2-H134R-EYFP expression within and between groups. The most successful technique was injection of neonatal mice using the RWM injection technique, with significantly greater percentage of SGNs transduced overall compared to any other age or technique (p’s < 0.05; Kruskal–Wallis with Dunn’s method for post-hoc analysis, Fig. 1F).

### Round window membrane injection method (RWM)

The RWM injection method was performed in neonatal, juvenile and adult mice. The RWM was first pierced with a pulled borosilicate pipette to allow efflux of cochlear fluid (perilymph). In some mice, the efflux slowed or stopped within a few minutes but for others, the perilymph continued to flow out even after 10 min. The viral vector was injected after 10 min regardless of efflux. In all cases there was leakage of the virus from the cochlea during and post-injection, as visualised by the fast green dye.

**Figure 2:**
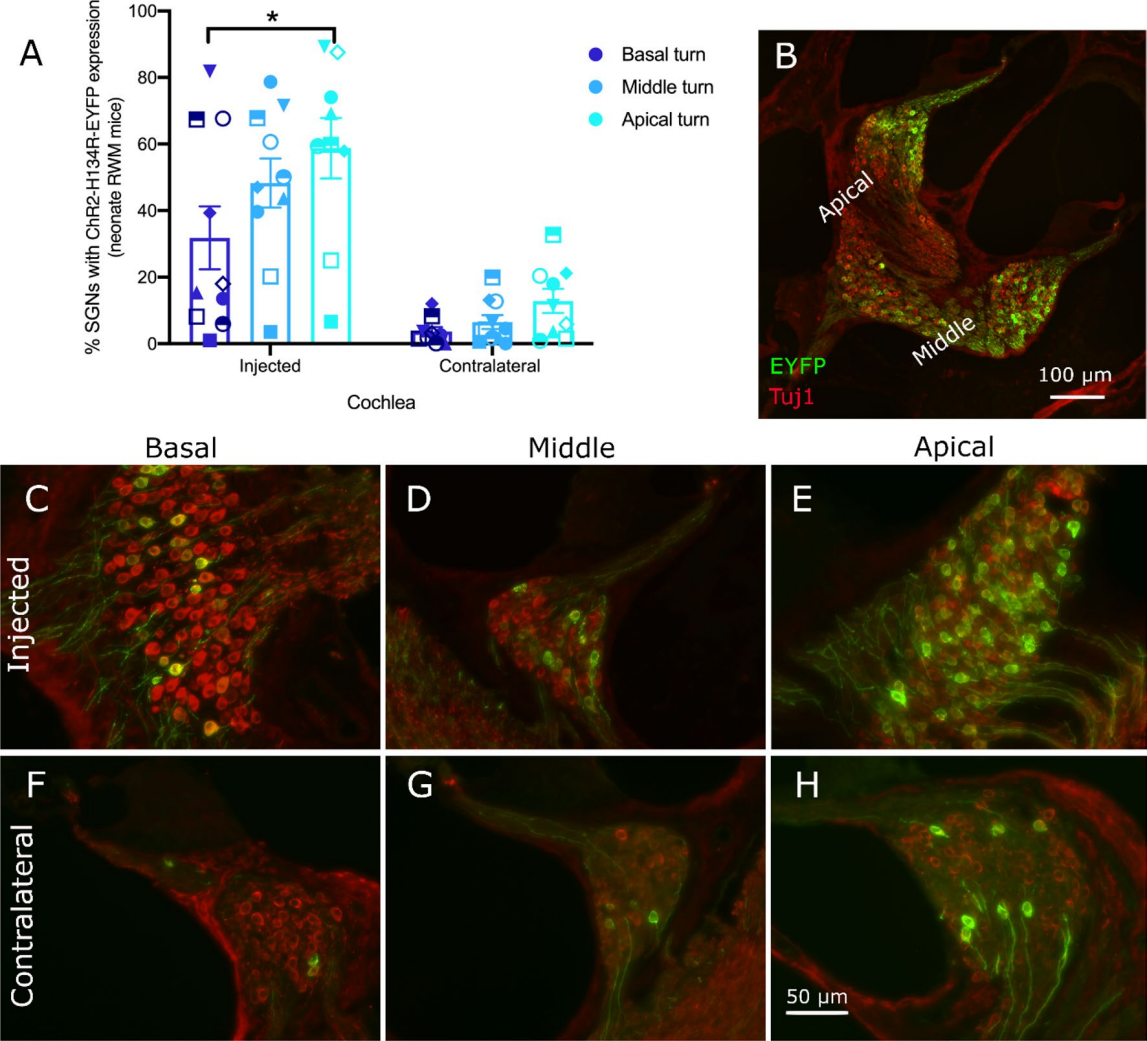
ChR2-H134R-EYFP expression in cochlear SGNs following RWM AAV injection in neonatal C57BL/6 mice. (**A**) The average percentage of EYFP-expressing SGNs was significantly different between each turn (p < 0.05; *p < 0.001 between base and apex; RM ANOVA; n = 10). A similar transduction pattern was observed on the contralateral side, but with overall lower transduction (error bars show the standard error of the mean; one mouse with no ChR2-H134R-EYFP expression was excluded in order to show average expression in successfully transduced mice). (**B**) Section of an injected cochlea showing the middle and apical turns. Transduced cells were visualised via the EYFP reporter (green) and the neurons were counterstained with the Tuj1 antibody (red). Expression of ChR2-H134R-EYFP was observed in SGN cell bodies and their central and peripheral fibres. (**C-E**) Images of Rosenthal’s canal from the basal, middle and apical turns of RWM-injected neonatal mice. The proportion of SGNs expressing ChR2-H134R-EYFP increased in a basal to apical direction. (**F–H**) Contralateral side Rosenthal’s canal images from the basal, middle and apical turns. Scale bar in H applies to images C to H. Minor image processing (brightness) was applied equally to the EYFP channel across C-H for representation purposes.

For neonatal C57BL/6 mice, injected at 3 ± 0.3 days old, 10 out of the 11 injected cochleae had ChR2-H134R-EYFP expression in SGNs when analysed 21–55 days post-injection (average 35 ± 3 days). Expression was in all turns of the cochlea, increasing from base to apex in all cases. Taking results from the 10 mice with ChR2-H134R-EYFP expression, average ChR2-H134R-EYFP expression in the basal, middle and apical turns was 32 ± 9%, 48 ± 8% and 59 ± 9% of total neurons in each particular turn, respectively. There was a significant difference between each turn (base and middle turns p = 0.043, middle and apical turns p = 0.026 and base and apical turns p < 0.001, RM ANOVA, n-10; Fig. 2A). The proportion of SGNs transduced was variable, with expression levels in the apex ranging from 7–89% and expression levels in the base ranging from 1–82% in the 10 successfully transduced mice. All mice with ChR2-H134R-EYFP expression on the injected side also had expression of ChR2-H134R-EYFP expression in SGNs in the contralateral non-injected cochlea that was, on average 13.9 ± 3.1 fold lower (p < 0.001, RM ANOVA, n = 10; Fig. 2A). Example images from the basal, middle or apical regions of injected and contralateral cochleae are shown in Fig. 2B-H.

In contrast, there were only two successfully transduced cochleae out of thirteen C57BL/6 adult mice injected via the RWM technique (47 ± 7 days old; range 24–101 days old). Cochleae were analysed after a post-injection period of 20–82 days (average 29 ± 5 days). The proportion of transduced SGNs in these two mice was 71% in the apical turn (only one data point available), 14 ± 2% in the middle turn but only 0.6 ± 0.05% in the basal turn. The remaining mice had no detectable EYFP with the exception of the occasional isolated EYFP-expressing SGN. The adult transduced mice also had expression in inner hair cells, while neonatal mice did not. The contralateral non-injected cochleae from adult mice had no detectable EYFP signal.

There were no EYFP-positive SGNs in the three juvenile mice injected with the RWM method (13–15 days old), although this group was small (n = 3) and was not pursued in detail.

### Round window membrane injection with SCC fenestration (RWM-F)

Six adult C57BL/6 mice were injected via the RWM with fenestration of the posterior semi-circular canal (average 28 ± 1 days old; range 25–34 days old). For this procedure, only minor perilymph efflux from the RWM was observed when the membrane was pierced which was wicked away prior to injection. The viral vector was then injected by hand (n = 3 mice) or a micro-pump over 30–60 s (n = 3 mice). Egress of the fast green dye from the SCC was confirmed in all cases indicating perfusion throughout the cochlea and semi-circular canals. There was no leakage of the virus from the RWM and green dye was clearly observed within the cochlea.

**Figure 3:**
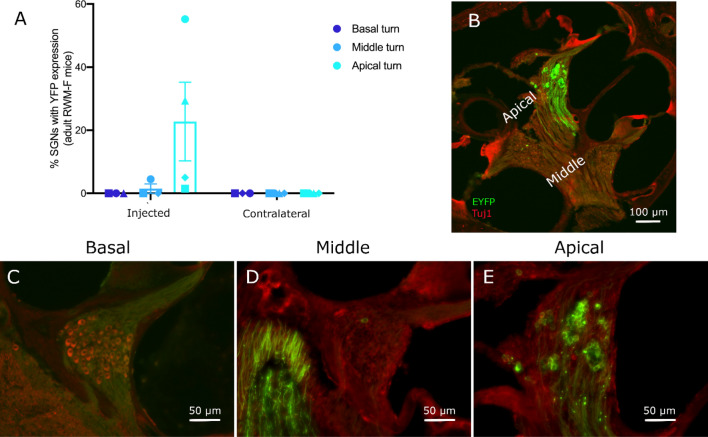
Expression of ChR2-H134R in cochlear SGNs following AAV-injection via the cochlear RWM of an adult C57BL/6 mouse with fenestration of the SCC (RWM-F). (**A**) The percentage of EYFP-expressing SGNs was highest in the apex but dropped dramatically by the middle turn and was not observed in the basal turn (n = 4; data excludes two mice in which there was no detectable ChR2-H134R-EYFP expression; error bars show the standard error of the mean). EYFP-expressing neurons were not observed in contralateral cochleae. (**B**) Section of an injected cochlea showing the middle and apical turns. Expression of ChR2-H134R-EYFP was observed in apical turn SGN cell bodies and their central and peripheral fibres. (**C-E**) Images of Rosenthal’s canal from the basal, middle and apical turn of an adult mouse injected via the RWM-F technique. Transduced cells were visualised via the EYFP reporter (green) and the neurons were counterstained with the Tuj1 antibody (red). The proportion of SGNs expressing ChR2-H134R-EYFP was high in the apical turn only. In the middles turn (**D**), EYFP-expressing central axons of SGNs from the apical turn are visible. Minor image processing (brightness) was applied equally to the EYFP channel across C-E for representation purposes.

Injected mouse cochleae were examined after 21–58 days (average 36 ± 5 days). Four out of the 6 injected cochleae had ChR2-H134R-EYFP expression, which was largely confined to the apical turn. Of these mice, the proportion of transduced neurons in the apical turn expressing ChR2-H134R-EYFP ranged from 5–55% of total apical SGNs (Fig. 3) and three were from the group injected by hand. Only one of these mice displayed expression in the middle turn in which only 5% of the neurons were transduced. ChR2-H134R-EYFP expression was not detected in the basal turn of any mouse. No detectable signal was detected in contralateral non-injected cochleae from the adult mice injected with the RWM-F technique.

Six adult guinea pigs (60 days old at the time of injection; range 57–64) were also injected via the RWM-F method. Two guinea pigs had an additional hole drilled into the modiolus to allow access of the virus into the neural tissue. Guinea pig cochleae were analysed after 20–41 days (average 33 days) but none had detectable EYFP expression.

### Semi-circular canal injection (SCC)

Four adult C57BL/6 mice were injected via the posterior semi-circular canal. Following opening of the semi-circular canal, perilymph efflux was observed and was wicked away prior to injection. The injection pipette was sealed in place so it was not possible to observe the injection via the fast green dye, but there was very little leakage of green dye observed following removal of the pipette. There was no expression of ChR2-H134R observed in the SGNs and no responses to optical stimuli were recorded in the inferior colliculus.

### Multiunit activity in the inferior colliculus

**Figure 4:**
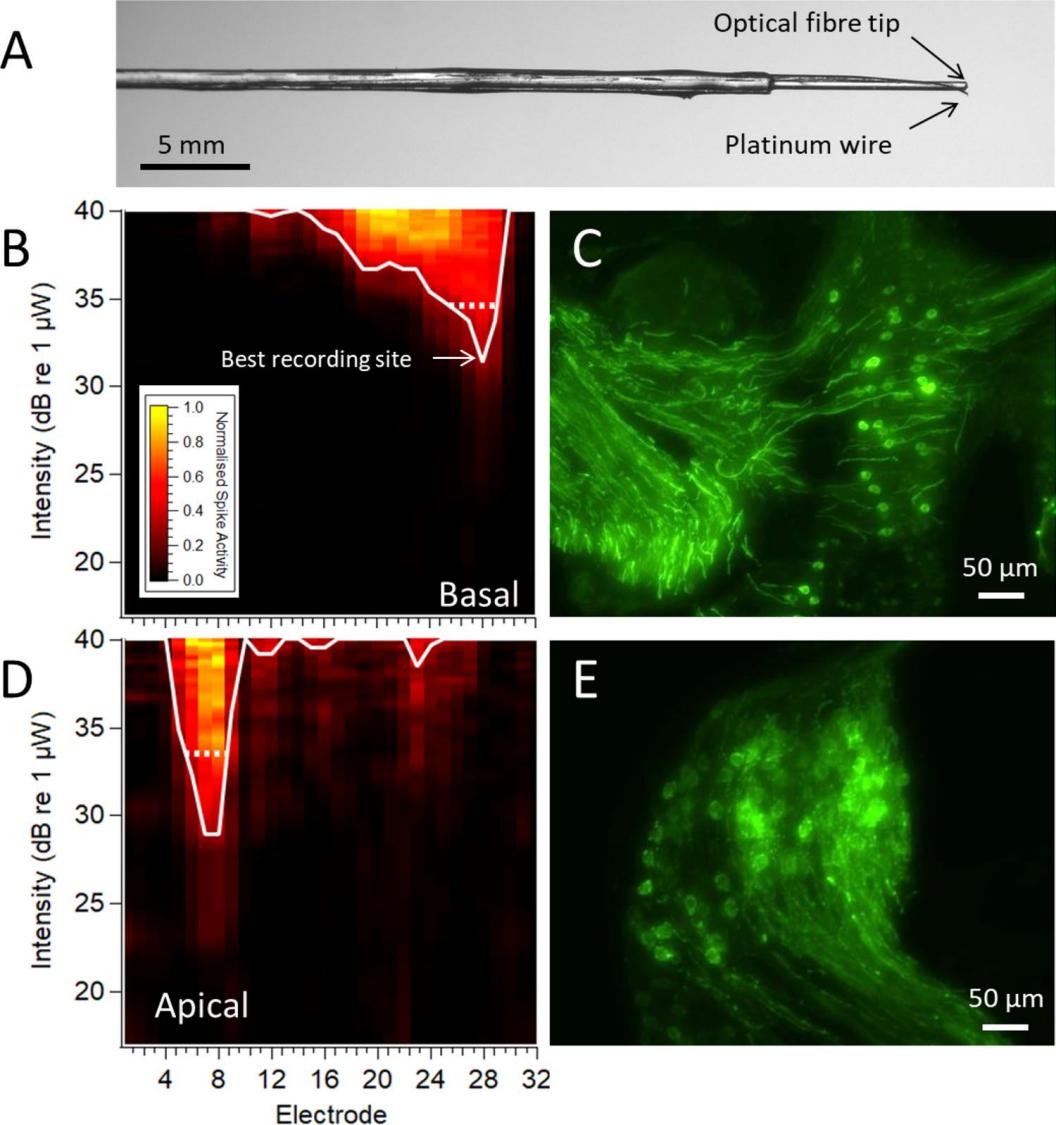
Cochleotopic multi-unit responses. (**A**) Optical fibre with bonded platinum wire. (**B**) Spatial response image to 1 ms optical pulses from a round-window positioned optical fibre in a neonatally AAV-injected C57BL/6 mouse. Low threshold multi-unit activity was recorded at ventral recording sites of the recording array in the inferior colliculus. Expression of ChR2-H134R-EYFP was observed in approximately 15–44% of total SGNs in the basal-mid region of the cochlea in this example, with the basal turn shown in (**C**). (**D**) Responses to apical optical stimuli were recorded in the more dorsal electrodes of the recording array in the inferior colliculus. Approximately 69% of total apical SGNs displayed ChR2-H134R-EYFP expression in this example (**E**). Each response image shows the spatial extent and normalised rate of multi-unit activity across the recording array which corresponds to depth in the inferior colliculus. Intensities were smoothed and normalized between spontaneous (black) and maximum rate (yellow). The white line represents the spatial tuning curve with threshold set to 30% of maximum activity. The dotted lines compare the width of activation at an equivalent level above threshold (d’ = 1 above threshold). Colour map in A applies to both response images. Minor image processing (brightness, contrast) was applied to images for representation purposes.

The inferior colliculus is the major midbrain structure where nearly all ascending auditory inputs converge with preservation of frequency representation that can be probed with a linear multichannel recording array. We performed inferior colliculus recordings in a subset of mice (neonatal- and adult-transduced C57BL/6 mice and ChR2-H134R-EYFP transgenic mice). Optical stimuli were applied to the cochlea via insertion of an optical fibre 1–2 mm through the RWM at the base of the cochlea or via a cochleostomy near the apex of the cochlea. The forward-emitting optical fibre was predicted to deliver light broadly to the basal-mid region of the cochlea, which best corresponds to the middle turn in our histological sections (as the basal turn was defined as the hook-basal turn region). A 25 µm platinum/iridium wire was bonded to the optical fibre to deliver electrical stimuli to the same position in the cochlea (Fig. 4A).

For the neonate group, multiunit activity in response to optical stimuli was recorded in five out of five mice with ChR2-H134R-EYFP expression that ranged from 6–82% in the base and 59–89% transduction in the apex. Responses to optical stimuli were detected in a cochleotopic manner, such that low-threshold responses were recorded at the more ventral recording sites within the inferior colliculus for RWM-positioned optical stimuli while more dorsal responses were recorded for optical stimuli applied to the apex of the cochlea via a cochleostomy (Fig. 4B-E).

**Figure 5:**
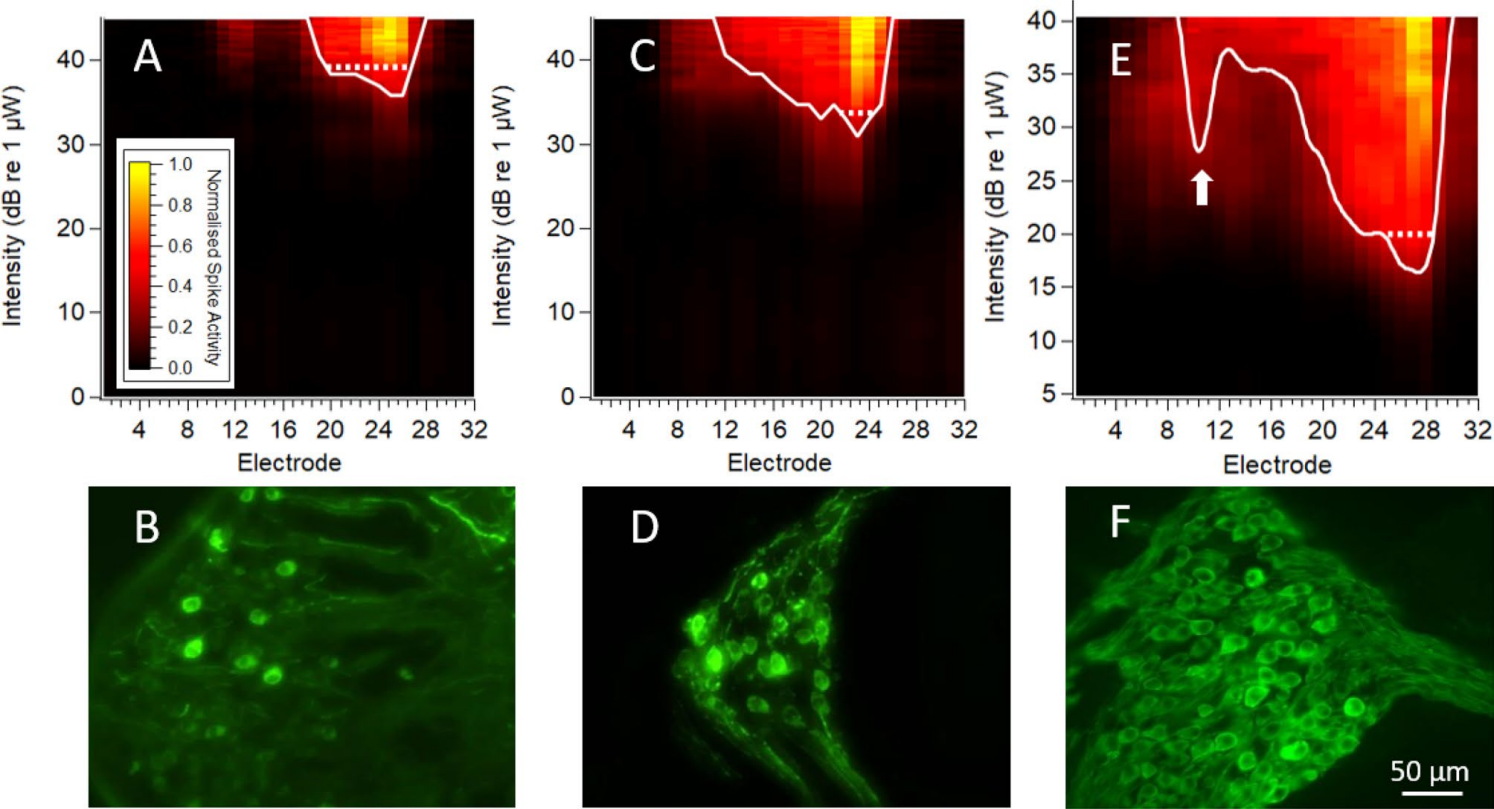
Responses to optical stimulation via a RWM-positioned optical fibre (neonatally AAV-injected C57BL/6 and ChR2-H134R-EYFP transgenic mice) (**A**) Spatial response images showing multi-unit activity at ventral recording sites in response to 1 ms optical stimulation applied via the RWM of the cochlea in a mouse with 6–40% ChR2-H134R-EYFP expression in the basal-mid turn region. (**B**) Image of Rosenthal’s canal from the basal turn of the mouse recorded in (A) showing ChR2-H134R-EYFP expression in occasional SGNs. (**C**) Multi-unit responses to RWM-applied optical stimulation recorded from a mouse with 72–82% of SGNs expressing ChR2-H134R-EYFP in the basal-mid turn region, as shown by the basal turn image of Rosenthal’s canal in (**D**). (**E**) Multi-unit responses to optical stimulation in a ChR2-H134R-EYFP transgenic mouse. A second area of activation at the more dorsal recording sites (arrow) can be observed indicating activation of apical SGNs from an optical fibre positioned in the RWM. This image has been reproduced with permission from^35^. (**F**) Image of the basal turn region Rosenthal’s canal from the ChR2-H134R-EYFP transgenic mouse with expression of in all SGNs. Scale bar in F applies to all histological images. Response images were generated as per Fig. 4. The dotted lines compare the width of activation at an equivalent level above threshold (d’ = 1 above threshold). Minor image processing (brightness, contrast) was applied to images for representation purposes.

**Figure 6:**
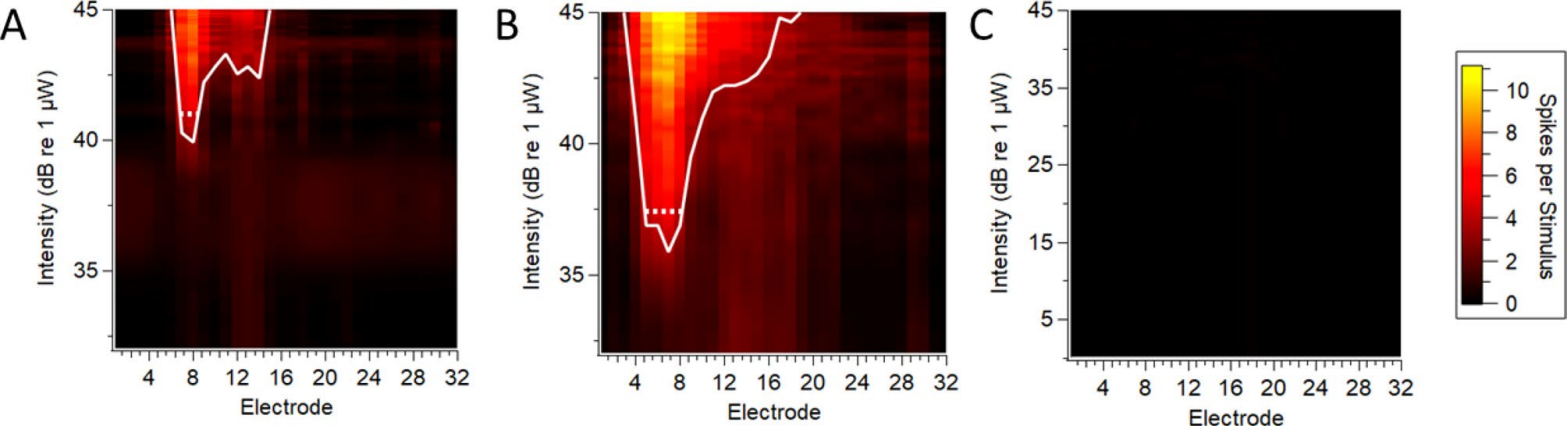
(**A**) Spatial response image recorded from 5 ms optical pulses from a RWM-positioned optical fibre in an AAV-injected C57BL/6 mouse with 55% ChR2-H134R-EYFP transduced cells in the apical turn but no basal turn expression (expression pattern shown in Fig. 3). The dorsal best electrode in this response image is suggestive of activation of apical turn neurons (**B**) Response image from the same mouse generated from an apically-positioned optical fibre (5 ms pulse). (**C**) No multiunit inferior colliculus activity was recorded in a mouse that had no detectable expression of ChR2-H134R-EYFP in any cochlear turn (image from 10 ms optical pulse). Response images were generated as per Fig. 4. The dotted lines compare the width of activation at an equivalent level above threshold (d’ = 1 above threshold).

Figure 5A-D show example response images to optical stimuli from a RWM-positioned fibre in mice with a low or a high proportion of transduced SGNs. There was a lower optical activation threshold for the mouse with high transduction in the basal-mid region of the cochlea. For comparison, a response image from a ChR2-H134R-EYFP transgenic mouse with opsin expression in all SGNs^35^ is shown in Fig. 5 E–F. In addition to the main area of response around the best electrode, there was often additional high threshold activity at the dorsal recording sites of the inferior colliculus, indicating apical turn SGN activation from the stimuli applied via a RWM-positioned optical fibre. This was especially evident in ChR2-H134R-EYFP transgenic mice (Fig. 5E) as well as the C57BL/6 mouse from the adult RWM-F group which had 55% transduced cells in the apical turn, but no expression in the basal turn (Fig. 6A). For the latter mouse, the best electrodes were almost identical regardless of whether the optical fibre was positioned in the RWM or apical turn, but the threshold for activation was 2.5 times higher when the optical fibre was in the RWM of the cochlea (Fig. 6A-B).

No activity was detected in the single mouse from the neonatal AAV-injected group that did not have detectable EYFP expression in any turn (Fig. 6C). In this case, the position of the recording electrode was confirmed via multiunit activity recordings from electrical stimulation (data not shown). Recordings from two other mice in the RWM-F group also exhibited responses to electrical but not optical stimulation; these mice had no expression in the base while one had only 5% expression in the apex. No optical responses were detected in mice from the semi-circular canal injection group (n = 3 recordings) or from mice from the adult RWM group (n = 2 recordings), none of which had detectable ChR2-H134R-EYFP expression and all of which had electrical responses (data not shown).

### Response thresholds and spread of activation in the cochlea

**Figure 7:**
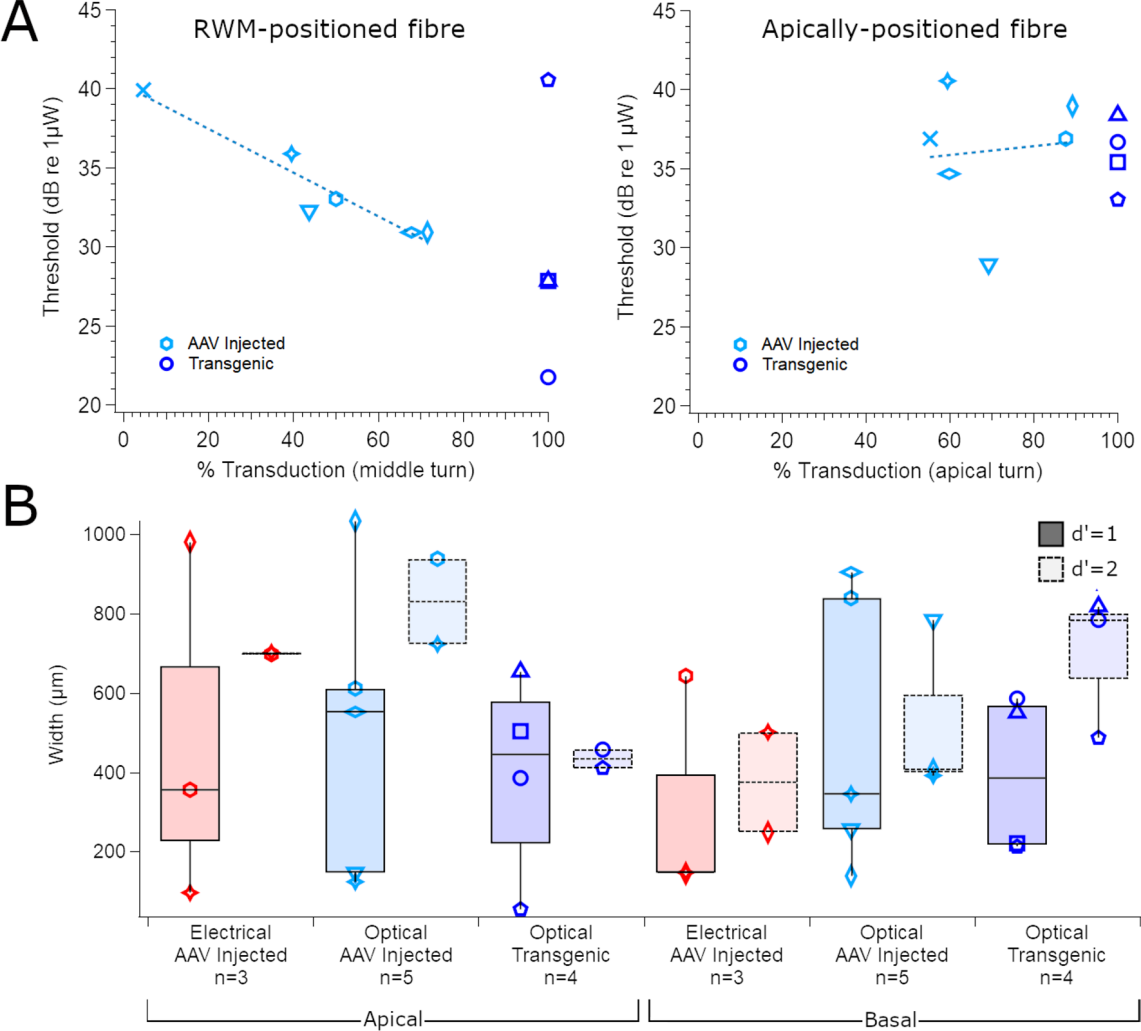
(**A**) Thresholds for optical activation via a RWM-positioned optical fibre or an apically-positioned optical fibre in C57BL/6 mice from the neonate AAV-injection group plotted against the percentage of neurons expressing ChR2-H134R-EYP in the middle turn (left; R^2^ = 0.91, p = 0.003) or apical turn (right; R^2^ = 0.19, p = 0.99) (n = 6). ChR2-H134R-EYFP transgenic mice with expression in all SGNs (100% of SGNs expressing ChR2-H134R-EYFP) are also shown for comparison (dark blue; n = 4). (**B**) Spatial tuning curve widths for electrical (red) or optical (blue) stimulation applied to the RWM or the apex of the cochlea. The width of the spatial tuning curve was measured at d’ = 1 above threshold or d’ = 2 above threshold where available. Spread of activation for transgenic mice are shown for comparison (darker blue). Box and whisker plots show median, first and third quartiles and the minima and maxima. Individual mice are symbol-coded. Basal turn data from ChR2-H134R-EYFP mice are a subset of the data presented in^35^.

Data is presented from 6 neonatally AAV-injected C57BL/6 mice with a range of transduction efficiencies from 0–82% transduction in the basal-mid turn region. When light was delivered through the RWM, the optical activation thresholds ranged from 30.9 to 39.9 dB re 1 µW (1.2 – 9.8 mW; n = 6). There was a correlation between optical activation threshold and the proportion of transduced SGNs in the middle turn (p = 0.003; R^2^ = 0.91; Fig. 7A), but not the basal turn (p = 0.077; R^2^ = 0.58), suggesting that the light from the forward-emitting optical fibre activates neurons towards the middle turn of the cochlea, as defined by histological sections. There was no significant correlation between transduction in the apex and optical response thresholds from the apically-positioned optical fibre (R^2^ = 0.19; p = 0.99), but there was a relatively narrow range (59–89%) in the percentage of transduced neurons in the apex (n = 6). The range for optical activation in the apex was 34.7 – 40.5 dB re 1 µW (2.92 – 11.3 mW). On average, the optical activation threshold was 4.05 dB higher for an apically-positioned optical fibre compared to the RWM-placement. By comparison, electrical activation thresholds were 0.24 dB higher in the apex compared to the base.

The spread of activation was measured at intensity levels of d’ = 1 above threshold, as well as d’ = 2 above threshold where possible. There was no significant difference in the spread of activation between optical-only and electrical-only stimulation at either the RWM or apex of the cochlea (two-way ANOVA (Mode, Region), n = 3, p = 0.177; Fig. 7B). Transgenic mice, that express ChR2-H134R-EYFP in all cochlear neurons, are shown for comparison.

### Hybrid stimulation

**Figure 8:**
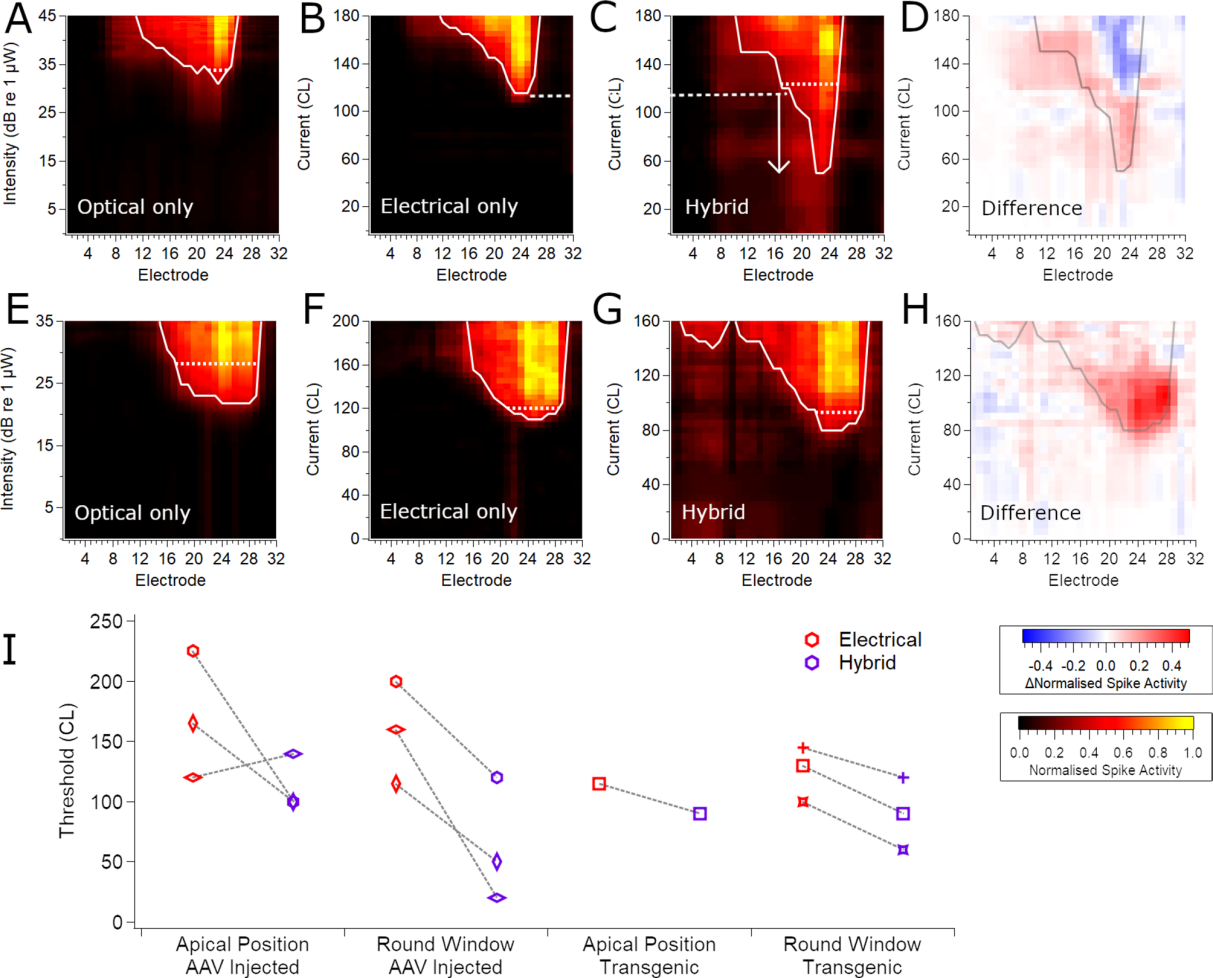
Interaction between electrical and optical stimuli reduces electrical thresholds. Example response images from a C57BL/6 neonatally AAV-injected mouse (**A-D**; up to 82% ChR2-H134R-EYFP transduced cells in the basal-mid turn region) or a ChR2-H134R-EYFP transgenic mouse (**E–H**) showing the spatial extent and rate of multi-unit activity across the recording array in response to RWM-applied 1 ms optical pulses (**A, E**), 100 µs/phase electrical pulses (**B, F**) or hybrid stimulation for which the electrical pulse was delayed by 1.75 ms relative to the optical pulse which was sub-threshold (63% of threshold optical power) (**C, G**). The dashed line/arrow between B and C shows the reduction in threshold afforded by the subthreshold optical stimulus applied prior to the electrical stimulation. (**D, H**) Difference plots to highlight activity that was greater or lower than the sum of the individual optical and electrical recordings in A-B or E–F. Response images were generated as per Fig. 4. The dotted lines on each spatial tuning curve compare the width of activation at an equivalent level above threshold (d’ = 1 above threshold). (**I**) Electrical thresholds for electrical stimulation alone (red) or when a subthreshold optical pulse was applied 1.75 ms prior to the electrical pulse (hybrid stimuli; purple) in the apex and RWM of the cochlea (n = 3 for injected mice and n = 1–3 for transgenic mice; a dashed line links data from the same mouse; individual mice are symbol-coded). Individual mice are symbol-coded. Basal turn data from ChR2-H134R-EYFP mice are a subset of the data presented in^35^.

Hybrid stimulation (the combination of sub-threshold optical and electrical stimuli) was also applied at the RWM and apical stimulation sites. The threshold for optical-only stimulation at the RWM or apex of the cochlea of C57BL/6 neonatally AAV-injected mice was first determined from the spatial tuning curves (Fig. 8A). Subsequently, electrical-only (Fig. 8B) or hybrid stimuli (using a sub-threshold level of optical stimulation; Fig. 8C) were applied to the same stimulation sites. For the RWM stimulation site in this example, each 1 ms optical pulse was at 63% of threshold power and was applied with an electrical pulse that was delayed by 1.75 ms, resulting in a reduction in the threshold for activation with electrical current by 65 CL (5.1 dB). The difference in the response images between electrical-only and hybrid stimulation was analysed and plotted to highlight areas where activity was greater than the sum of electrical-only and optical-only stimulation. Figure 8D shows that the increased activity can be observed as a sharp peak in the tuning curve near the best electrode. For the apical turn, the optical power was at 75% of threshold and also reduced electrical threshold by 65 CL (5.1 dB; data not shown). Similar interactions were observed for the other mice analysed except for the RWM stimulation site of one mouse in which there was only 6% SGNs transduced with ChR2-H134R-EYFP near the stimulation site (n = 4 mice from the neonate RWM injection group for which a complete data set for optical/electrical/hybrid stimulation were available). Interaction between optical and electrical stimulation was seen with as little as 24% of threshold optical power. For comparison, data from RWM stimulation site in a ChR2-H134R-EYFP transgenic mouse is shown in Fig. 8E-H.

When subthreshold optical stimulation was applied to the RWM of the cochlea (applied 1.75 ms prior to the electrical pulse at 30–75% of threshold power for light stimuli), the threshold for electrical activation was, on average, 39 ± 20% of the normal electrical threshold (p = 0.071, paired T-test; n = 3). Subthreshold optical stimulation in the apical turn (at 55–75% of threshold power, 1.75 ms prior to the electrical pulse) also reduced the electrical thresholds in two out of three cases (n = 3; Fig. 8I). Data from ChR2-H134R-EYFP transgenic mice is shown for comparison. Hybrid stimulation did not have any consistent effect on spread of activation for AAV-injected mice and in many cases the best electrode differed between optical and electrical stimulation (data not shown).

## Discussion

We describe three methods of delivering viral vectors into the cochlear perilymphatic fluids for the expression of the ChR2-H134R opsin in SGNs and subsequent optical activation of these neurons. Optical responses, tested in a subset of mice, were recorded in all mice in which ChR2-H134R-EYFP expression was detected, except where less than 5% of total SGNs were transduced. The threshold of neural activation correlated with the proportion of transduced SGNs for RWM-applied optical stimulation, but not for apical turn stimulation. Animals with a predominantly apical pattern of opsin expression could effectively be activated from an optical fibre positioned in the RWM, either by transmission of light through the tissue or activation of central axons of apical neurons that pass the basal turn. These results provide a basis for understanding the impact of opsin expression in the cochlea for novel neural modulation techniques such as optical or hybrid stimulation.

### Opsin expression

The most successful method for SGN transduction was injection of viral vectors via the RWM in neonatal mice. In agreement with our observations, injection of Anc80, AAV2/6, AAV-PHP.B or AAV-ie viral vectors via the RWM of neonatal mice is well documented to result in expression of transgenes in the majority of SGNs (~ 60–80%), often with a trend of higher expression in the apex as well as contralateral spread^22,23,25,40^. Our longest experimental timeline was 89 days following injection of the viral vector, whereupon expression of ChR2-H134R-EYFP was still apparent. Long-term expression has been observed in many studies^21,23^, with the longest time point examined to date being 9 months in mice^25^. We found transduction of contralateral SGNs to be limited to neonatal mice which is also in agreement with other studies^21^.

By contrast, the same RWM injection technique in adult mice was unsuccessful in most cases. Low transduction rates and high variability in transduction in adult mice may be a consequence of developmental maturation changes to the tissue as well as the efflux of perilymphatic fluid that occurs following the opening of the RWM during injection. Cerebrospinal fluid flows into the cochlea via the cochlear aqueduct to maintain fluid homeostasis^41^ and tends to wash out the injected virus resulting in highly variable dosing. To overcome this, fenestration of the posterior semi-circular canal was used in this study and others to encourage flow of the virus through the cochlea and resulted in very high eGFP expression (via a CMV promoter) in inner and outer hair cells throughout the cochlea^38^ and improved the number mice (but not guinea pigs) with SGN transduction. pihIt did not alter the overall proportion of transduced neurons or the gradient of expression in adult mice, which was still high in the apex and absent in the basal turn. This base-apex gradient of transduction has been previously reported for AAV2/6 driving expression of the ChR2 variant CatCh via the synapsin promoter^24^ and could be a reflection of differential activity of the synapsin promoter between the base and apex in adult mice. Indeed, longitudinal differences in molecular expression characteristics of spiral ganglion neurons and hair cells along the tonotopic axis of the cochlea have been reported^42–44^. Alternatively there may be variability of access of the viral vector to the SGN membrane which becomes highly myelinated from the onset of hearing^45^.

Injection via the posterior semi-circular canal is an attractive alternative to intracochlear injection, as it leaves the delicate cochlear tissue intact and has been shown to preserve hearing thresholds in adult animals^46–50^. In adult CBA/CaJ mice, injection of the Anc80 virus containing the eGFP gene (CMV promoter) into the posterior semi-circular canal resulted in GFP expression in nearly 100% of hair cells and also up to 10% of SGNs^50^. However, from 4 injected mice we did not detect any EYFP-expressing SGNs with the Anc80 virus injected via the posterior semi-circular canal, despite successfully using this technique for transducing hair cells and supporting cells with a reporter gene using an Anc80 viral vector with a CMV promoter (data not shown).

Another method used to transduce SGNs with opsins has been direct injection into the spiral ganglion in adult gerbils^21,26,51^. This method was developed due to very low transduction rates following injection into the perilymph of the scala tympani in the gerbil, as we found for guinea pigs in this study. AAV2/6 or AAB-PHP.B were used to express CatCh or f-Chrimson via the synapsin promoter. A pressure micro-injector was used to inject the viral vector suspension directly into the spiral ganglion which requires delicate drilling into the modiolar bone and resulted in some loss of SGNs, but nonetheless resulted in CatCh/f-Chrimson expression in 50–90% of injected adult gerbils and optical responses in many of those animals^21,26,51^. Although we also drilled into the modiolus in two guinea pigs in this study, we did not use a pressure micro-injector and it is possible that the delivery of the AAV suspension to the modiolus was not sufficient to result in transduction.

The synapsin promoter was used with the intention to restrict opsin expression to SGNs, but we found that in adult mice there was additional expression in inner hair cells. For proof-of-concept studies it is important to eliminate possible hair cell-mediated optical responses by using methods of hair cell inactivation, regardless of opsin expression, such as acute neomycin exposure as was used here. Off-target expression in hair cells and supporting cells of another type of opsin, Chronos, has been reported following Anc80-mediated gene transfer in 4 day old mice via a CAG promoter^23^. Similarly, some transgenic mouse models of opsin expression in SGNs also have expression in inner and outer hair cells, due to the use of the parvalbumin promoter^35^, but displayed no adverse effects of opsin expression on hair cell function.

This and other studies have shown that species and age can have a significant impact on transduction of cell types and as such it is difficult to extrapolate the findings to humans. In adult cynomolgus monkeys, AAV9-PHP.B was injected into the cochlea via the RWM. There was dose-dependent transduction of hair cells and SGNs throughout the cochlea suggesting that clinical therapy will be possible using a safe injection technique^52^. More specifically to channelrhodopsin, ChR2 was safely expressed over many months in the frontal cortex of the macaque via lentivirus-mediated gene transfer and optical activation was possible^53^. Collectively, these studies highlight the potential for clinical translation of optogenetic techniques for neural activation in the cochlea.

### Functional responses

Expression of ChR2-H134R-EYFP in > 5% SGNs was required for responses to the optical stimulation parameters used in this study. When light was applied to the cochlea through the RWM, lower optical thresholds correlated with a higher proportion of transduced SGNs counted in the middle turn from histological sections, reflecting the probable site of peak activation from the forward emitting optical fibre. A similar correlation of optical activation to the proportion of opsin-transduced SGNs has been recently reported for f-Chrimson in the Mongolian gerbil^51^. Additionally, it has been found that increased expression of ChR2 within individual SGNs increases excitability of SGNs^37^. In our study, there was no correlation between optical thresholds and electrical thresholds, suggesting that inter-animal differences in optical thresholds were not due to differences in distance between the stimulating hybrid device and the SGNs. When light was applied to the apical turn via a cochleostomy, no correlation between optical thresholds and transduction levels was observed. However, a lower range in transduction and possible variability in the way that the hybrid optical/electrical device was inserted through the apical turn cochleostomy may have prevented the observation of any trend in the data.

A typical spatial tuning curve generated from a RWM-positioned optical fibre in the neonatally-injected mice had low threshold multi-unit activity in the ventral recording sites of the inferior colliculus, but also high threshold activity at the more dorsal recording electrodes. This phenomenon is in keeping with our observations in transgenic mice that express ChR2-H134R-EYFP in all SGNs (see Fig. 5F)^35^. In contrast, the unique apical-only expression pattern in the adult-injected mice resulted in multi-unit activity only at dorsal recording sites from a RWM-positioned optical fibre, providing direct evidence of high threshold apical neuron activation from the RWM of the cochlea. There are two possible reasons for this response pattern. There may be activation of SGN central axons that originate from the apex of the cochlea at the point where they pass the basal turn as they express ChR2-H134R-EYFP ion channels along with the peripheral fibre and cell body. Another possibility is that some light from the tip-emitting optical fibre used in this study passes through the relatively thin mouse cochlear tissue at the higher light intensities and activates the apical neurons, as postulated previously^21,35^. Optimising the light path towards the modiolus, such as via an array of micro-light emitting diodes^54^, would increase the efficiency of neural activation and may therefore reduce the spread of activation.

To directly compare optical and electrical stimuli within each mouse, we used a hybrid device where the optical fibre and platinum wire were bonded together. Widths of the spatial tuning curve measured along the tonotopic axis of the inferior colliculus (representative of spread of activation in the cochlea) during stimulation via an optical fibre in mice injected with Anc80-ChR2-H134R-EYFP were not significantly different to activation with electrical current from a 25 µm platinum/iridium wire, as we found previously for ChR2-H134R-EYFP transgenic mice^35^. It should be noted that the size of the platinum wire used for electrical activation is significantly smaller than is possible for clinical devices as it would create a charge density too high for chronic electrical stimulation and result in eventual platinum dissolution^55^. As such, it represents a best-case scenario for spread of activation from electrical stimulation rather than the broader spread normally observed from platinum electrode rings in mice^26,56^. It was also not possible to optimise the direction of the light from the forward-emitting optical fibre due to the anatomical limitations in the mouse. Thus, the spread of activation that we observed from optical activation in the Anc80-opsin transduced mice was not as narrow as previously reported for the AAV-2/6 or AAV-PHP.B-opsin transduced gerbil^26^. The thickness/density of tissue may also permit more transmission of light beyond the immediate site of stimulation in the mouse.

When sub-threshold optical and electrical stimuli were combined as hybrid stimuli (with 1.75 ms delay between the optical and electrical pulse), we observed reduced electrical activation thresholds during stimulation via the RWM-positioned device, although measurements were only possible in 3 mice and therefore reduced the power of statistical analysis. Reduced electrical thresholds will, in turn, reduce current spread, as we observed in ChR2-H134R-EYFP transgenic mice where hybrid stimulation reduced activation thresholds and reduced the spread of activation compared to electrical-only stimulation and optical-only stimulation^35^. However, a reduction in the spread of activation in viral-mediated opsin-transduced mice did not occur consistently. This may be explained by the fact that the individual optical and electrical stimuli did not always activate the same region of the cochlea due to the pattern of opsin expression in the neurons. A case in point is shown in Fig. 6 where the apical ChR2-H134R-EYFP expression pattern resulted in apical activation in the cochlea despite the placement of the optical fibre in the basal turn, reducing the opportunity for the optical and electrical stimuli to interact. Overall, our data provide further support that hybrid stimulation can increase the spectral resolution of activation in the cochlea.

### Clinical translation

Efficiency and specificity of opsin expression will need to be improved prior to future clinical development, with particular emphasis on the testing of injection routes, AAV tropism and specific promoters in non-human primates in which the anatomy of the cochlea and gene expression profiles of the SGNs are most similar to humans. Stability of opsin expression over time will also need to be considered. While long-term opsin expression has been demonstrated in some neural tissues^53^, specific studies in the cochlea have not yet been performed. Expression of opsins in the contralateral cochlea was observed for neonatally injected mice in this study and is likely to be the result of an increase in perilymphatic pressure within the non-pliant otic capsule of the cochlea during injection of the viral vector, forcing perilymph out of the cochlea via the cochlear aqueduct^41^. Contralateral expression of transgenes following AAV injection is variable between species and method of injection but it does highlight the need to test for off-target expression and potentially adverse effects in the brain and other tissues. There have been no studies in the cochlea examining the impact of long-term implantation such as fibrous tissue formation around optical emitters, which has long been an issue for electrical activation thresholds and hearing outcomes in recipients of electrical cochlear implants^57^. In this study, the direction of the light emission was not optimised. A clinical device would likely require a method to direct the light towards the modiolus, such as thin film arrays of light-emitting diodes that have been demonstrated to efficiently activate opsin-expressing SGNs in rodents^58,59^. Multichannel devices such as these will be necessary to determine whether hybrid stimulation can reduce channel interactions which would be required to enhance hearing outcomes with the cochlear implant. Retaining electrical stimulation in hybrid devices means that the technology is backwards compatible (i.e. a recipient could use electrical-only stimulation if desired or required) which may aid in clinical acceptability and translation by reducing the higher risk associated with optical-only devices.

## Conclusions

This study demonstrates that both optical-only and hybrid stimulation are significantly influenced by the proportion of SGNs expressing opsins as well as the overall expression pattern in the cochlea. As such, the development of techniques for highly efficient, cochlear-wide expression of opsins will be required for clinical application of optical-only or hybrid cochlear implants (the combination of optical and electrical stimuli where one or both modes are used at sub-threshold levels). Optical-only stimulation did not improve the spread of activation compared to electrical-only stimulation with the parameters used in this study, but a beneficial interaction between optical and electrical stimuli was demonstrated, supporting the use of hybrid stimulation technology to improve the way neurons are modulated for the treatment of sensory deficits or other neurological conditions. The project outcomes have the potential to improve the listening experience of cochlear implant recipients, but also to be applied to other areas of neural modulation where high precision is required.
